# Anti-Actin IgA Antibodies Identify Celiac Disease Patients with a Marsh 3 Intestinal Damage among Subjects with Moderate Anti-TG2 Levels

**DOI:** 10.1155/2013/630463

**Published:** 2013-09-05

**Authors:** Enrico Schirru, Fabrice Danjou, Lucia Cicotto, Rossano Rossino, Maria Doloretta Macis, Rosanna Lampis, Rita-Désirée Jores, Mauro Congia

**Affiliations:** ^1^Department of Public Health, University of Cagliari, Cittadella Universitaria, Monserrato, 09045 Cagliari, Italy; ^2^Gastroenterology Unit, Microcitemico Hospital, ASL8 Cagliari, Via Jenner, 09121 Cagliari, Italy

## Abstract

A new diagnostic tool (algorithm-1) for coeliac disease (CD) permitting the diagnosis without performing the duodenal biopsy has been recently proposed by the European Society for Paediatric Gastroenterology, Hepatology, and Nutrition (ESPGHAN). It combines symptoms associated with CD, high anti-transglutaminase type 2 antibody (anti-TG2) levels, anti-endomysium-IgA antibodies (EMA), and at-risk HLA. Our aims were (i) to evaluate retrospectively in 227 individuals (149 CD patients and 78 controls) the algorithm-1, (ii) to reduce the number of duodenal biopsies among CD patients for whom algorithm-1 is not applicable through the addition of antiactin IgA antibodies (AAA-IgA), and (iii) to evaluate prospectively algorithm-1 and AAA-IgA in 50 patients with suspected CD. Algorithm-1 identified 70 out of 149 CD patients with Marsh 3 lesions. Adding AAA-IgA to the remaining patients with anti-TG2 levels comprised between 4 and 10 times upper limit of normal (ULN) allowed the detection of further 20 patients with a Marsh 3 damage. In our prospective study, algorithm-1 identified 23 out of 50 patients, whilst further 7 were recognized adding AAA-IgA. We confirm that algorithm-1 may avoid the duodenal biopsy in many CD patients and underscores the usefulness of AAA-IgA in reducing the number of duodenal biopsies in patients with moderate anti-TG2 levels.

## 1. Introduction

Coeliac disease (CD) is an immune-mediated systemic disease, triggered and maintained by dietary gluten in genetically predisposed individuals, characterized by a variable small intestinal villous damage and by different clinical manifestations [1]. 

Recently, a synopsis summarizing some of the evidence statements and recommendations of the guidelines in CD diagnosis for use in clinical practice has been formulated by a working group within the European Society for Paediatric Gastroenterology, Hepatology, and Nutrition (ESPGHAN) [2]. 

An important statement of these guidelines is the development of two new algorithms for CD diagnosis based on (i) the presence of symptoms and signs suggestive of CD in children and adolescents (algorithm-1) and (ii) the absence of symptoms and signs in persons at genetic risk for developing CD (algorithm-2). 

We have considered in the present work the most interesting of the two new algorithms, algorithm-1. It allows diagnosis of CD without performing the duodenal biopsy in children and adolescents with symptoms and signs suggestive of CD, anti-transglutaminase type 2 antibody (anti-TG2) levels >10 times upper limit of normal (ULN), and positive confirmation tests of anti-endomysium-IgA antibodies (EMA) and with the presence of at-risk HLA-DQ2 or -DQ8. If all these requirements are fulfilled, the diagnosis of CD is confirmed, gluten-free diet is started, and the patient is studied for improvement of symptoms and decline of autoantibodies. A later gluten challenge in these patients is not required [2]. 

However, it has been established that symptomatic CD patients with elevated degrees of intestinal damage may also have anti-TG2 levels lower than 10 times ULN [3, 4]. Therefore, a high number of symptomatic CD patients with anti-TG2 levels lower than 10 times ULN, and to whom algorithm-1 cannot be applied, still necessitate a duodenal biopsy. 

Since anti-actin IgA antibodies (AAA-IgA) directed against actin filaments are strongly correlated with total or subtotal intestinal atrophy [5, 6], we hypothesized that serum measurement of this autoantibody may contribute in increasing the number of patients who can avoid a duodenal biopsy. 

Aims of this study were (i) to evaluate retrospectively in 227 individuals (149 CD patients and 78 controls) the performance of algorithm-1, (ii) to reduce further the number of duodenal biopsies among CD patients in whom algorithm-1 cannot be applied with the addition of AAA-IgA, and (iii) to evaluate also prospectively the performance of algorithm-1 combined with AAA-IgA levels in 50 individuals with symptoms suggestive of CD. 

## 2. Material and Methods

### 2.1. Patients

Our group consisted of 163 consecutive Sardinian CD patients (121 females, 42 males, ratio females/males 2.9, mean age at diagnosis 8 years, and range from 2 to 18 years); 149 presented symptoms suggestive of CD (Table 1), whilst 11 were not included in the study because they were asymptomatic and, for this reason, not belonging to the algorithm-1. Also, three patients with IgA deficiency, a well-known condition complicating the interpretation of the serological pattern of CD, were excluded. All patients were diagnosed according to ESPGHAN criteria [7] and were recruited from subjects attending the ambulatory of the Pediatric Gastroenterological Unit in Cagliari, Italy, between 2005 and 2012. A further group of 78 individuals with persistent significant gastrointestinal symptoms, already characterized by upper digestive endoscopy and small bowel biopsy [6], were used as non-CD subjects. All 227 individuals were characterized for histopathology, anti-TG2, EMA, HLA typing, and AAA-IgA.

An additional group of 50 patients with symptoms suggestive of CD according to the new ESPGHAN criteria [2] were prospectively diagnosed to evaluate the ability of the combination of algorithm-1 plus AAA-IgA to further reduce the number of intestinal biopsies (Table 1). 

Informed consent was obtained from subjects (or from their parents if minor) participating in the prospective study.

### 2.2. Anti-TG2

Anti-TG2 was dosed using the ELIA commercial kit-ImmunoCAP (Phadia, Milan, Italy) after serum dilution when necessary. Results were expressed in times upper limit of normal with a cutoff of 7 U/mL. 

### 2.3. EMA

EMA was determined by immunofluorescence (Delta Biologicals, Rome, Italy). Results were expressed by intensity of immunofluorescence from 0 to 4. With the aim of reducing false positives, only a strong intensity of immunofluorescence (2 or higher) was considered positive. 

### 2.4. HLA Typing

To type for HLA-DRB1 and DQB1 alleles [8], HLA Olerup SSP Molecular Typing Kits (Roche, Sweden) were used according to the manufacturer's instructions. The identification of the various DRB1, DQA1, and DQB1 haplotypes in CD patients was performed following the segregation of HLA haplotypes in families [9]. In this study, the two forms of HLA-DQ2, termed DQ2.5 and DQ2.2, respectively, were considered separate because the risk of coeliac disease conferred by DQ2.2 is lower than that conferred by DQ2.5, unless it is expressed together with DQ2.5 [10, 11].

### 2.5. AAA-IgA

AAA-IgA is an immunofluorescence serological test developed in our laboratory that has been validated in a multicenter study [5, 6] as a useful marker of an elevated grade of intestinal damage associated with CD. Results were expressed by intensity of immunofluorescence from 0 to 4. With the aim of reducing false positives, only a strong intensity of immunofluorescence (2 or higher) was considered positive.

### 2.6. Histopathology

For each patient, 2 or more biopsies were taken from the second/third portion of the duodenum (at least a minimum number of 4 samples), and at least 1 or more biopsies were taken from the duodenal bulb [2, 12]. Intestinal villous atrophy has been graded according to the Marsh classification [13], modified by Oberhuber in types 3c, 3b, and 3a, 2, 1, and 0 [14], always by the same board-certified pathologists. 

### 2.7. Statistical Analysis

The frequencies of outcomes were analyzed using the chi-square test where appropriate. A *P* value < 0.05 was considered significant.

## 3. Results

**Figure 1:**
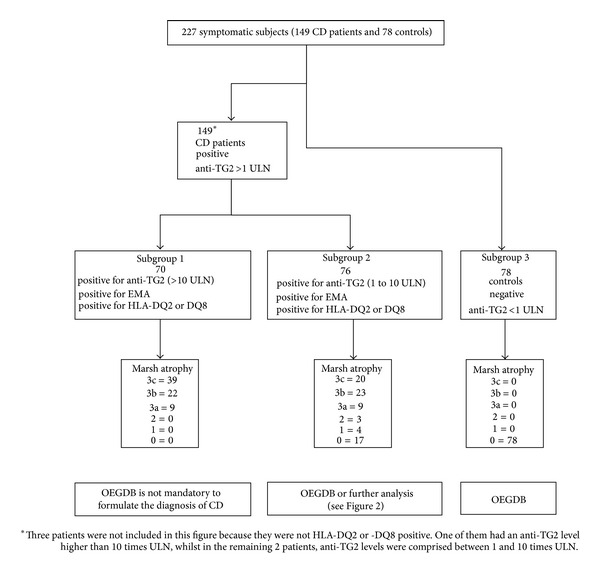
Distribution of CD patients and controls according to algorithm-1 in the retrospective study. For each of the 3 Subgroups, the degree of intestinal atrophy is also illustrated. CD: coeliac disease; anti-TG2: anti-transglutaminase type 2 antibody; ULN: upper limit of normal; EMA: anti-endomysial antibodies; HLA: human leukocyte antigen; 3a, 3b, 3c, 2, 1, and 0 indicate the grade of intestinal damage according to the Marsh-Oberhuber classification [13, 14]; OEGDB: oesophagogastroduodenoscopy and biopsy; and AAA-IgA: anti-actin IgA antibody.

The one hundred and forty-nine CD patients and 78 controls were classified in 3 different Subgroups according to algorithm-1 based on the presence of symptoms and signs suggestive of CD, anti-TG2 levels, positivity for EMA and for HLA-DQ2 (in *cis* or in *trans*), or -DQ8 (Figure 1). 

We found that all patients with symptoms and signs suggestive of CD, anti-TG2 levels >10 times ULN, positivity for EMA and for HLA-DQ2 (in *cis* or in *trans*), or -DQ8 had coeliac disease with a Marsh 3 atrophy (Subgroup 1 of Figure 1). This Subgroup consisted of 70 of the 227 subjects.

As far as the HLA associated risk was concerned, three out of the 227 subjects expressed the DQ2.2 molecule without DQ2.5. One of them had anti-TG2 levels higher than 10 times ULN and showed a Marsh 3c histopathology, whereas the remaining two patients had an anti-TG2 level under 10 times ULN and a Marsh 0. These patients were not considered further because the low number did not consent to draw any conclusion. 

Among the 227, seventy-six CD patients were included in Subgroup 2 that differed from Subgroup 1 only for the anti-TG2 levels lower than 10 times ULN (Figure 1). It is important to note that Subgroup 2 included 17 patients with potential CD since they were positive for CD specific antibodies and at-risk HLA but they did not show the histopathological changes associated with CD [2]. The remaining Subgroup 3 consisted of the seventy-eight control individuals (Figure 1).

Based on these data, the association of AAA-IgA with severe intestinal damage was evaluated in all three Subgroups. As expected since AAA-IgA is a marker of severe intestinal damage, a higher frequency of positive AAA-IgA was found in Subgroups 1 and 2 that included the CD patients compared to Subgroup 3 that included the controls. However, we found that in Subgroup 1, the addition of AAA-IgA to algorithm-1 did not offer any further useful information as only 76.5% of the CD patients, already defined by algorithm-1, had a positive AAA-IgA. 

**Figure 2:**
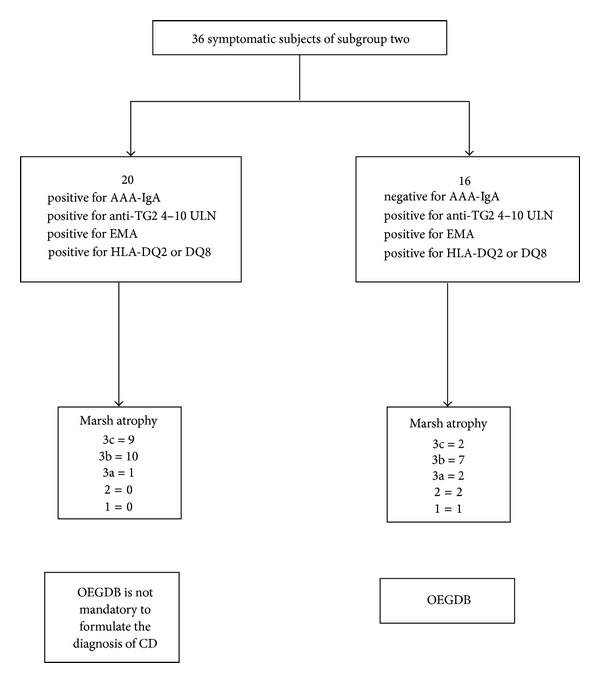
AAA-IgA measurement in subjects of Subgroup 2 with anti-TG2 levels comprised between 4 and 10 times ULN. Distribution of AAA-IgA in the 36 subjects of Subgroup 2 with anti-TG2 levels comprised between 4 and 10 times ULN. CD: coeliac disease; anti-TG2: anti-transglutaminase type 2 antibody; ULN: upper limit of normal; EMA: anti-endomysial antibodies; HLA: human leukocyte antigen; 3a, 3b, 3c, 2, 1, and 0 indicate the grade of intestinal damage according to the Marsh-Oberhuber classification [13, 14]; OEGDB: oesophagogastroduodenoscopy and biopsy; and AAA-IgA: anti-actin IgA antibody.

Conversely, the addition of AAA-IgA to Subgroup 2 increased the correlation with Marsh 3 histopathology (OR = 9.5; *P* < 0.05), and it became complete when the range of anti-TG2 comprised between 4 and 10 times ULN values was chosen. On the contrary, for values of anti-TG2 lower than 4 times ULN, a number of AAA-IgA false positives were observed (data not shown). Using this strategy, further 20 CD patients of Subgroup 2 with Marsh 3 histopathology (Figure 2) were identified. Finally, in Subgroup 3, only 3 out of 78 controls were AAA-IgA positive.

In the prospective study, twenty-three out of 50 patients were detected by algorithm-1 and the intestinal biopsy was not performed. For the remaining 27 subjects, the addition of AAA-IgA in those with anti-TG2 values comprised between 4 and 10 times ULN, allowed the detection of further seven CD patients with a Marsh 3 intestinal damage (Table 2). In patients with anti-TG2 levels lower than 4 times ULN, only an incomplete association between algorithm-1, plus AAA-IgA and Marsh 3 intestinal damage was found.

In all 50 patients, including the 23 CD diagnosed with the algorithm-1 and the 7 diagnosed with the addition of the AAA-IgA, gluten-free diet progressively caused a reduction of anti-TG2 levels and disappearance of the symptoms.

## 4. Discussion

The new algorithm-1 recently reported by ESPGHAN [2] suggested that in the diagnosis of CD, duodenal biopsies could be avoided in children and adolescents with CD associated symptoms, high anti-TG2 levels, and positivity for EMA and at-risk HLA. The aims of the present study were to confirm the reliability of algorithm-1 and to further reduce the number of duodenal biopsies by adding the serum measurement of another marker of intestinal damage (AAA-IgA) [6, 15, 16].

In our retrospective study, we found that the algorithm-1 would have avoided the duodenal biopsy in 70 out of 149 CD patients (Subgroup 1 of Figure 1). We also found that all the 70 CD patients had a Marsh 3 grade of intestinal damage. This finding confirms, as previously suggested, that anti-TG2 level >10 times ULN is strongly associated with the most severe intestinal damages [17, 18]. On the other hand, we noticed that algorithm-1 was not able to detect with sufficient accuracy a Marsh 3 damage when the values of anti-TG2 were lower than 10 times ULN. In fact, among the 76 subjects of Subgroup 2, differing from Subgroup 1 only for anti-TG2 levels less than 10 times ULN, a variable degree of intestinal damage ranging from Marsh 3 lesions to Marsh 0 was found.

Since numerous studies have reported a strong association of AAA-IgA with severe CD intestinal damage [6, 15, 16] and since the likelihood of CD is unequivocal when a villous atrophy of Marsh 3 is found [13, 14], we measured the AAA-IgA in Subgroups 1, 2, and 3, with the aim of identifying those subjects having Marsh 3 intestinal histopathology. 

The most important result was obtained by the measurement of AAA-IgA in Subgroup 2, where positivity for AAA-IgA and a Marsh 3 lesion was restricted to subjects having a level of anti-TG2 antibody comprised between 4 and 10 times ULN (Figure 2). This strategy allowed the identification of 20 additional CD patients with a Marsh 3 lesion (Figure 2) in whom the duodenal biopsies could have been avoided. 

Whereas a wide range of sensitivity and specificity values of AAA-IgA have been reported, questioning its utility in the screening for CD [19], our data suggest that the best usage of AAA-IgA measurement in the diagnosis of CD is not as an isolated test, but in association with anti-TG2 values comprised between 4 and 10 times ULN.

When the anti-TG2 antibody titer is higher than 10 times ULN, the association with a Marsh 3 lesion is complete, and no other test should be requested; low values of positivity for anti-TG2 are found also in non-CD patients affected by other autoimmune pathologies as well as infections, tumors, myocardial damage, liver disorders, and psoriasis [20, 21]. 

Our retrospective results were confirmed by our prospective study, in which a cohort of 50 patients with suspected CD underwent to algorithm-1. Indeed, all 7 AAA-IgA positive patients who had anti-TG2 values comprised between 4 and 10 times ULN showed a Marsh 3 intestinal damage.

## 5. Conclusions

Our findings confirm that the duodenal biopsy may be omitted in a significant number of CD patients by the application of algorithm-1. In addition, we show that positivity for AAA-IgA in children and adolescents with anti-TG2 antibody levels comprised between 4 and 10 times ULN may further reduce the number of duodenal biopsies.

## Figures and Tables

**Table 1:** Prevalence of the symptoms suggestive of CD in our retrospective (149 CD patients) and prospective (50 CD patients) studies.

Symptoms	Retrospective study	Prospective study
Diarrhoea	23.1%	21.3%
Iron deficiency anaemia	17.0%	16%
Short stature/growth failure	16.5%	12%
Abdominal pain	15.4%	9.3%
Weight loss	8.8%	10.7%
Chronic fatigue	5.5%	9.3%
Constipation	4.9%	6.7%
Vomiting	2.7%	5.3%
Increased level of liver enzymes	2.2%	2.7%
Irritability	1.1%	2.7%
Others	2.7%	4%

**Table 2:** Distribution of the intestinal damage in our prospective study according to AAA-IgA in patients with anti-TG2 levels comprised between 4 and 10 times ULN.

Serological tests	Intestinal atrophy according to Marsh/Oberhuber classification					
	3c	3b	3a	2	1	0
AAA-IgA+ and anti-TG2 4–10 ULN	5	1	1	0	0	0
AAA-IgA− and anti-TG2 4–10 ULN	1	3	2	0	1	1
